# Test-Retest Reliability of 10 Hz Conditioning Electrical Stimulation Inducing Long-Term Potentiation (LTP)-Like Pain Amplification in Humans

**DOI:** 10.1371/journal.pone.0161117

**Published:** 2016-08-16

**Authors:** Weiwei Xia, Carsten Dahl Mørch, Ole Kæseler Andersen

**Affiliations:** 1 Center for Neuroplasticity and Pain (CNAP), SMI ®, Department of Health Science and Technology, Aalborg University, Aalborg, Denmark; 2 China-Japan Union Hospital, Jilin University, Changchun, Jilin Province, China; University of Modena and Reggio Emilia, ITALY

## Abstract

**Background:**

10 Hz conditioning electrical stimulation (CES) has been shown to induce long-term potentiation (LTP)-like pain amplification similar to traditional 100 Hz CES in healthy humans. The aim of this study was to assess the test-retest reliability and to estimate sample sizes required for future crossover and parallel study designs.

**Methods:**

The 10 Hz paradigm (500 rectangular pulses lasting 50 s) was repeated on two separate days with one week interval in twenty volunteers. Perceptual intensities to single electrical stimulation (SES) at the conditioned skin site and to mechanical stimuli (pinprick and light stroking) in immediate vicinity to the conditioned skin site were recorded. Superficial blood flow (SBF) was assessed as indicator of neurogenic inflammation. All outcome measures were assessed with 10 min interval three times before and six times after the CES. The coefficient of variation and intra-class correlation coefficient were calculated within session and between sessions. Sample sizes were estimated for future crossover (Ncr) and parallel (Np) drug testing studies expected to detect a 30% decrease for the individual outcome measure following 10 Hz CES.

**Results:**

Perceptual intensity ratings to light stroking (Ncr = 2, Np = 33) and pinprick stimulation (491 mN) (Ncr = 6, Np = 54) increased after CES and showed better reliability in crossover than parallel design. The SBF increased after CES, and then declined until reaching a plateau 20 minutes postCES. SBF showed acceptable reliability both in crossover and parallel designs (Ncr = 3, Np = 13). Pain ratings to SES were reliable, but with large estimated sample sizes (Ncr = 634, Np = 11310) due to the minor pain amplification.

**Conclusions:**

The reliability of 10 Hz CES was acceptable in inducing LTP-like effects in the assessments of superficial blood flow, heterotopic mechanical hyperalgesia, and dysesthesia in terms of sample sizes for future crossover study designs.

## Introduction

Long-term potentiation (LTP) demonstrated as a long-lasting increased synaptic strength is an important feature of synaptic plasticity in the central nervous system [1,2]. LTP in hippocampus is supposed to be involved in learning and memory formation [2]. LTP of synaptic transmission in nociceptive pathways has been considered to be an underlying mechanism behind central sensitization [3–5]. Various conditioning electrical stimulation (CES) protocols have been used to induce LTP in the central nervous system *in vivo* and *in vitro* [6–9] and the perceptual correlates in pain on humans [10–16]. The CES induced LTP-like pain amplification in healthy humans has been considered to be a surrogate model of hyperalgesia and allodynia in patients [10,17,18]. Different frequencies of CES is intended to mimic different nociceptor discharging frequencies following tissue damage, e.g., 10 Hz CES mimic the low frequency discharging of C- fiber nociceptors during certain natural inflammatory pain conditions [19–21], whereas 100 Hz bursts mimic the initial high frequency discharging immediately after a tissue injury [20]. Among them, 10 Hz electrical stimulation has previously been shown to induce LTP of field potentials in the spinal dorsal horn by electrical stimulation of the tract of Lissauer which has a high percentage of small fibers transmitting nociceptive activity [6]. Similarly, 10 Hz CES of primary afferent fibers can induce LTP of spinothalamic tract neurons which were also involved in the nociception transmission [9]. In humans, continuous 10 Hz CES has been shown to induce long-lasting facilitation of perceived pain intensity to mechanical stimuli similar to the traditional high frequency (100 Hz) bursts CES [22]. Moreover, low frequency CES is associated with higher neurogenic superficial blood flow increase (neurogenic inflammation) than high frequency CES [22,23].

However, no studies so far have shown the reliability of 10 Hz CES induced pain amplification and inflammatory changes before potential translation of this human model to pharmacological testing or pain modulation studies. Test-retest reliability is an assessment of the measurement error (variation) that can be deemed to be acceptable. It is quantified by the extent to which the measurements are consistent [24,25]. The traditional reliability tests include assessments of relative and absolute reliability [26]. The relative reliability refers to the degree to which individuals maintain their position over repeated measurements usually by use of intra-class correlation coefficient (ICC); in contrast, the absolute reliability refers to the degree to which repeated measurements vary for individuals and is traditionally measured by the coefficient of variation (CV) and Bland-Altman analysis [26]. Additionally, sample size estimation has now been proposed to be an alternative to traditional measures of absolute and relative reliability [25,27,28].

The aim of the present study was 1) to measure the test-retest reliability of sensory and neurogenic inflammation measurements in 10 Hz CES paradigm within and between sessions, and 2) to estimate the sample sizes needed for mechanical and electrical stimulation outcome measures of hyperalgesia and dysesthesia, and imaging technologies used for assessing neurogenic inflammation. The sample size estimations can be used for future crossover and parallel pharmacological testing or pain modulation studies when employing the 10 Hz CES paradigm.

## Methods

### Subjects

The experiments were performed on 20 subjects (8 females and 12 males ranging in the age from 20 to 36 years; mean age 24 years) after obtaining approval from the Research Ethics Committee of the North Denmark (N-20120046). All subjects participated in a training session and two experimental sessions. During all sessions, the subjects were seated in a reclining chair with the right arm placed comfortably on the table in a room with temperature at 22~24°C. Exclusion criteria were prior or current skin disease, neurological disease, any history of chronic pain as well as drug abuse or suffering from ongoing pain. All subjects gave written informed consent prior to their inclusion in the study. The study was performed according to the Declaration of Helsinki.

### Conditioning Electrical Stimulation (CES)

**Fig 1:**
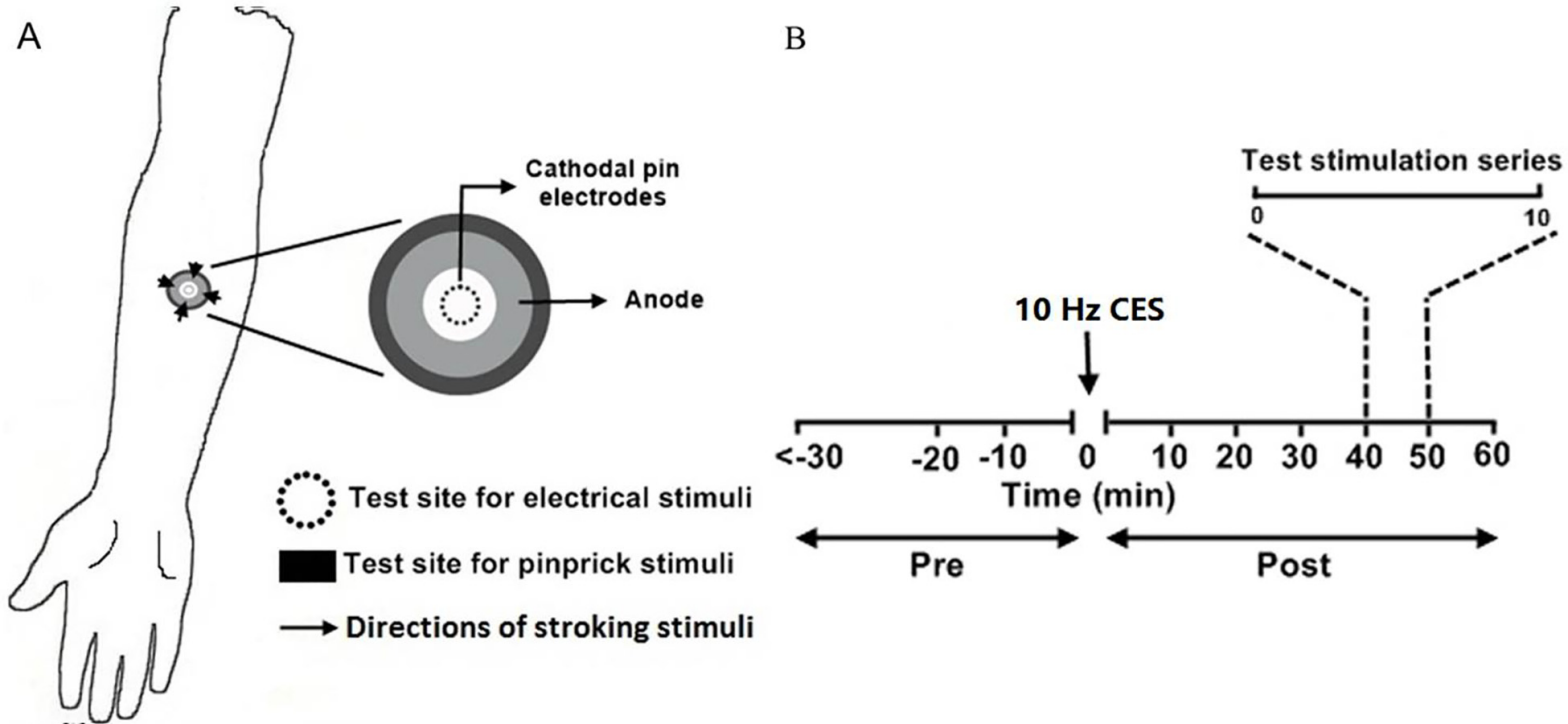
Experimental setup. (A) 10 Hz conditioning electrical stimulation was applied at the volar forearm 7 cm distal to the cubital fossa via a circular array of pin electrodes in two sessions. The single electrical stimulation was applied at the conditioned site by the same pin electrodes and the light stroking and pinprick stimuli were applied in the surrounding skin area. (B) Test stimulation series including SBF, ST, pain perception rating to pinprick and light-stroking stimuli, HPT and pain perception rating to SES was assessed with 10 min intervals three times before (pre-conditioning period) and six times after the CES (post-conditioning period). SBF: superficial blood flow; ST: skin temperature; HPT: heat pain threshold; SES: single electrical stimulation.

Cutaneous electrical stimulation from a constant current stimulator (DS5; Digitimer Ltd; Welwyn Garden City, UK) was applied to the right forearm 7cm distal to the cubital fossa. The electrical stimuli were applied using an epicutaneous pin electrode (EPE) consisting of a circular array (diameter: 10 mm; area: 79 mm^2^) of fifteen cathodal electrodes each with a diameter of 0.2 mm, protruding 1 mm from the base, and a large circular stainless steel plate served as anode with an inner diameter of 20 mm and an outer diameter of 40 mm placed concentrically around the cathodes (Fig 1*A*) (Biurrun Manresa *et al*., 2010). This electrode has been verified to induce pain/stinging at low stimulation intensities compared with conventional cutaneous patch electrodes because the diameter of the cathodes is smaller so high current density is achieved in the epidermal layers where the nociceptive Aδ- and C-fibers terminate (Mouraux et al., 2010; Mørch et al., 2011). The individual electrical detection threshold (DTh) was determined using the method of limits: three series of electrical pulses with increasing and decreasing stimulus intensities at a step size of the 3% present stimulation intensity. In each series of stimulation pulses, all subjects stopped the stimulation when feeling the electrical pulse upon increasing the intensities, and then stopped again when the electrical pulse became insensible upon decreasing the intensities. The electrical stimulus intensities were recorded as the DTh each time they stopped. The final DTh was determined by the geometric mean of the six DThs in three series of assessments. 10 Hz CES (pulse duration: 1 ms) was used for induction of LTP-like pain. This CES process lasted 50 sec and consisted of 500 rectangular 1 ms pulses applied (continuous train, no bursting) at 10× DTh evoking a clearly painful sensation.

### Experiment Protocol

Three sessions were arranged for each subject. The first training session was aimed to familiarize the subjects with the different stimulus modalities and gaining experience with rating these stimuli. This arrangement helps to reduce any systematic errors, i.e., learning effects [29]. The data obtained during the training session was not analyzed further. The 10 Hz CES paradigm for inducing LTP-like pain was repeated on two different days separated by at least one week for the individual subject. All subjects were blinded throughout the study with respect to the sequence of the pinprick stimuli and rationale of the study. A set of outcome measures similar to the study by Xia et al., 2016 was applied at the right forearm three times before and six times after the CES with 10 min interval [22] (Fig 1*B*). The assessments consisted of neurogenic inflammation imaging using laser speckle blood-flow imagery for measuring superficial blood flow (SBF) and infrared thermography for measuring skin temperature (ST), assessments of pinprick and light-stroking perception intensities surrounding the conditioned site (heterotopic), perceived intensity to homotopic single electrical stimulation (SES) and heat pain threshold (HPT) at the conditioned site. To evaluate perceived stimulation intensity, a VAS was used that was anchored from 0 (no sensation) to 100 (the most intense pain imaginable) where 30 indicated the pain threshold [28,30–32]. The same researcher performed all experiments to rule out the inter-rater variation.

### Perception Features of CES

The subjects were asked to rate continuously the magnitude of pain induced by the 10 Hz CES using a hand-held VAS device. These VAS ratings were sampled by a computer. All subjects were further asked to describe the quality of the CES using the short-form McGill Pain Questionnaire (SF-MPQ). The SF-MPQ consists of sensory (S) and affective (A) dimensions of pain, evaluative overall intensity of total pain experience (E), and present pain intensity (PPI) index of the standard MPQ. PPI is the average pain intensity of the 10 Hz CES that all subjects gave after finishing rating the conditioning process. All rating scores were added together to get a total quantitative value (Melzack, 1987).

### Neurogenic Inflammation

To assess the possible excitation of peptidergic nerve fibers and observe the temporal changes of SBF in the area surrounding the conditioned site, a Full-Field Laser Perfusion Imager (FLPI) was used to assess the SBF index (MoorFLPI; Moor Instruments Ltd, Axminister, Uk). The changes in ST in the area surrounding the conditioned site were measured using infrared thermography (Thermovision A40; FLIR; Danderyd, Sweden). The SBF and ST were measured in a round area with a diameter of 15 mm concentric to the circular pin electrodes. The measured area did not cover the area of pinprick stimuli.

### Light-Stroking Stimuli

A cotton swab was used for light stroking stimuli (~100mN) for assessing the tactile perception around the conditioned site. The stroking was performed in four test areas moving from the outer region towards the center of the conditioning electrodes (Fig 1*A*). The subjects gave a perception rating to the light-stroking with a distance of 1 cm moving at a speed of 1–2 cm/s using the VAS as mentioned above. The stroking stopped at 1 cm to the border of circular pin electrodes. An average of the four tests was used as the perception intensities for light-stroking stimuli.

### Pinprick Stimuli

Mechanical pinprick-evoked perception was assessed by three custom made weighted pinprick stimulators (125 mN, 294 mN, 491 mN; rounded tip, 0.2 mm in diameter, contact time 1~2 s; SMI®, Aalborg University) which were randomly applied in the area adjacent to the conditioned site (i.e. at 1.5–2 cm distance to the border of the cathodal pin electrodes) (Fig 1*A*). The subjects indicated the perceived intensity on the same VAS.

### Heat Pain Threshold (HPT)

The heat pain threshold was measured using a thermode placed at the conditioned site (Pathway; 30×30mm ATS; Medoc Ltd.; Ramat Yishai, Israel). The baseline temperature was 32°C, and the temperature was increased at a rate of 1°C/s until the subject indicated the perception of heat pain on a response button. Subsequently, the temperature returned to the baseline at a rate of 8°C/s. An average of three tests was used as the heat pain threshold.

### Single Electrical Stimulation (SES)

A single rectangular 1 ms constant-current electrical stimulation (intensity: 10×DTh) was applied as a homotopic electrical test stimulus using the same conditioning electrode placed at the conditioned site (Fig 1*A*). The subject rated the perceived intensity using the same VAS. An average of three tests with 10 sec interval was used as the pain intensity to SES at the conditioned site.

### Data Evaluation and Statistics

For evaluating the temporal changes of pain intensity during the 10 Hz CES process, the highest VAS rating in each 10 sec was chosen, i.e., five VAS ratings throughout the 50 sec conditioning period. The data (pre- and post- CES) was included in the statistical analysis. The VAS ratings of light-stroking stimuli and the SBF index were logarithmically transformed to obtain the lognormal distribution. The one-way repeated measure analysis of variance (RM-ANOVA; SPSS v. 21.0) was used to determine differences between the SF-MPQ scores of CES in two sessions. The two-way RM-ANOVA (the time and conditioning sessions were within-subjects factors) was used for SBF, ST, HPT, pain perception intensities to light-stroking and pinprick stimuli, and SES to determine the systematic bias between two sessions and the effect size of the CES. Greenhouse-Geissers method was used for the correction of non-sphericity. Bonferroni-Holm adjustment was used for multiple comparisons. The data is presented as mean values±SEM (standard error of the mean). *P*-value<0.05 was considered to be statistically significant.

The relative reliability was assessed using intra-class correlation coefficient (ICC) whereas the absolute reliability was assessed using coefficient of variation (CV) and Bland-Altman analysis. These three methods are most commonly used to report reliability in test-retest studies up to date [25,28,33]. Moreover, the 95% confidence interval (CI) of the ICC and CV in crossover and parallel study design was presented. Sample size estimations for crossover and parallel study designs were also estimated as a valid alternative approach for assessing the reliability in the present study.

The ICC two-way mixed model (type: consistency) was used to estimate the relative reliability both within and between sessions. The reliability related to the consistency of a test or measurement was quantified by ICC which is variation between subjects divided by the total variation in the data [34]. The ICC ranges from 0 (no agreement) to 1 (perfect agreement). Even though there is no consensus on the cut-point values, values above 0.75 are usually suggested to indicate good reliability and below 0.75 to indicate fair to poor reliability [35]. The ICCs of single measures for crossover (ICCwi) and parallel (ICCbt) study design were reported. Coefficient of variation (CV) was used for estimating the absolute reliability and is the standard deviation (SD) divided by the mean (*μ*) of each measurement for each individual. Bland-Altman analysis is an evaluation method for analyzing the limits of agreement of measurements after CES in two experimental sessions. This method estimates the interval in which 95% of the differences (*d*) between two measurements lies, i.e., the average measurement difference $(\bar{d})\pm 1.96\times \text{SD}$ of the measurement difference. The 95% confidence interval (CI) of bias and agreement limits was calculated for observing the precision of estimated limits of agreement. The 95% CI of bias illustrates the systematic difference. It was calculated as $\bar{d}\pm \;t\times \sqrt{{SD}^{2}/n}$ where the t value is 2.09 with 19 (n-1) degree of freedom at t distribution, and *SD* is the standard deviation of the differences. If the level of equality (i.e., the mean difference is zero) is not in the CI, a significant systematic difference between two sessions should be considered. In addition, the 95% CI of $\bar{d}\pm 1.96\times \text{SD}$ was calculated as the observed values± t×$\sqrt{{3SD}^{2}/n}$. The limits of agreement provide an estimation of the sample error. The sample sizes were calculated for a parallel and a crossover study. The desired significance level (α) was set to 0.05, and the desired power (1-β) was set to 0.8. The clinically relevant effect (*E*) was estimated to be 30% difference between baseline measurements and average value of the post-conditioning measurements in the first 30 min after CES. For a parallel study design, the sample size (Np) was estimated as: Nparallel = (15.6×σ^2^)/E^2^ where σ is the average value of the standard deviation of the post-conditioning measurements in the first 30 min. For a crossover study design, the sample size (Ncr) was estimated as: Ncrossover = (15.6×σ^2^×(1-ICC))/E^2^ where ICC was calculated from within sessions [25,36].

## Results

The average DTh was 186±71 μA across the two sessions (mean±SD, n = 40). No difference was found for the DTh between the two sessions. The CES intensity was 1.86±0.71 mA (10×DTh, mean±SD, n = 40). Most subjects perceived this intensity as painful (36±15.8) as shown in the SES measurement (preCES, n = 40). No visible skin injuries occurred following the electrical stimulation in any of the sessions.

### Perception Features of 10 Hz CES

**Fig 2:**
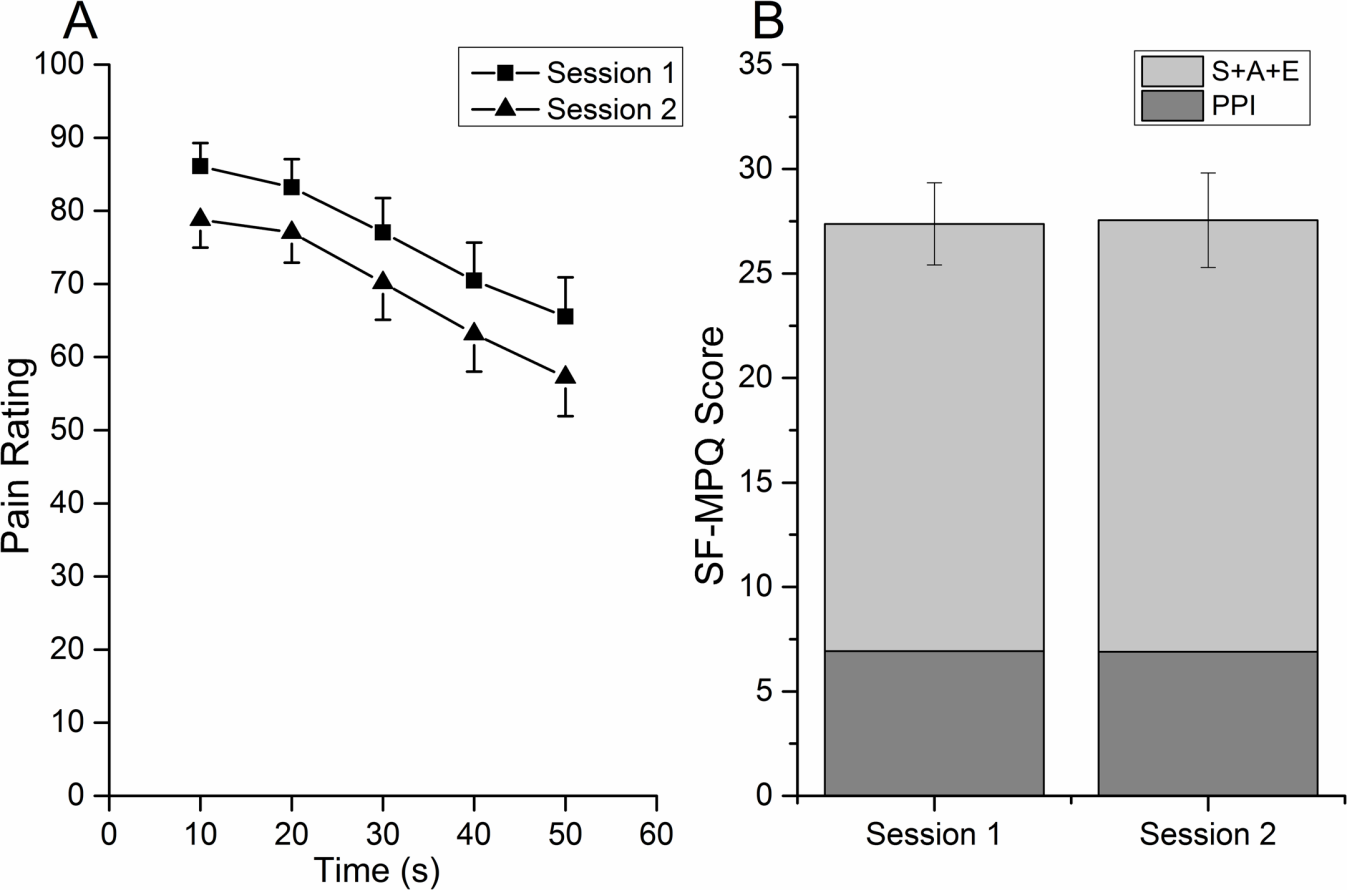
Pain experience induced by CES. A. Temporal changes during the conditioning process. The highest pain rating in each 10 s was chosen to compare the two sessions. The pain intensities declined in both sessions. B. Depiction of SF-MPQ scores for CES. The SF-MPQ scores and PPI were not found significantly different between the two sessions. PPI: present pain intensity; S: sensory; A: affective; E: evaluative overall intensity of total pain experience.

In the two sessions, the perception during CES gradually declined (time effect, F = 24.04, p<0.01; Fig 2*A*), i.e., the perception intensity in the first (0–10 s) and the second (10–20 s) 10 s was higher than the third (20–30 s), fourth (30–40 s) and fifth (40–50 s) pain rating (p<0.05, Bonferroni-Holm); the perception intensity in the third 10 s was higher than fourth and fifth pain rating (p<0.01, Bonferroni-Holm); the perception intensity in the fourth 10 s was higher than the fifth pain rating (p<0.01, Bonferroni-Holm). The pain perception in session one was higher than in session two (session effect, F = 7.36, p<0.05) (Fig 2*A*). The SF-MPQ scores were not found to be significantly different between the two sessions (F = 0.011, p = 0.92) (Fig 2*B*). The PPI for the conditioning process in the two sessions was not significantly different (F = 0.009, p = 0.926). No interaction was found between session and time factors.

### Neurogenic Inflammation

**Fig 3:**
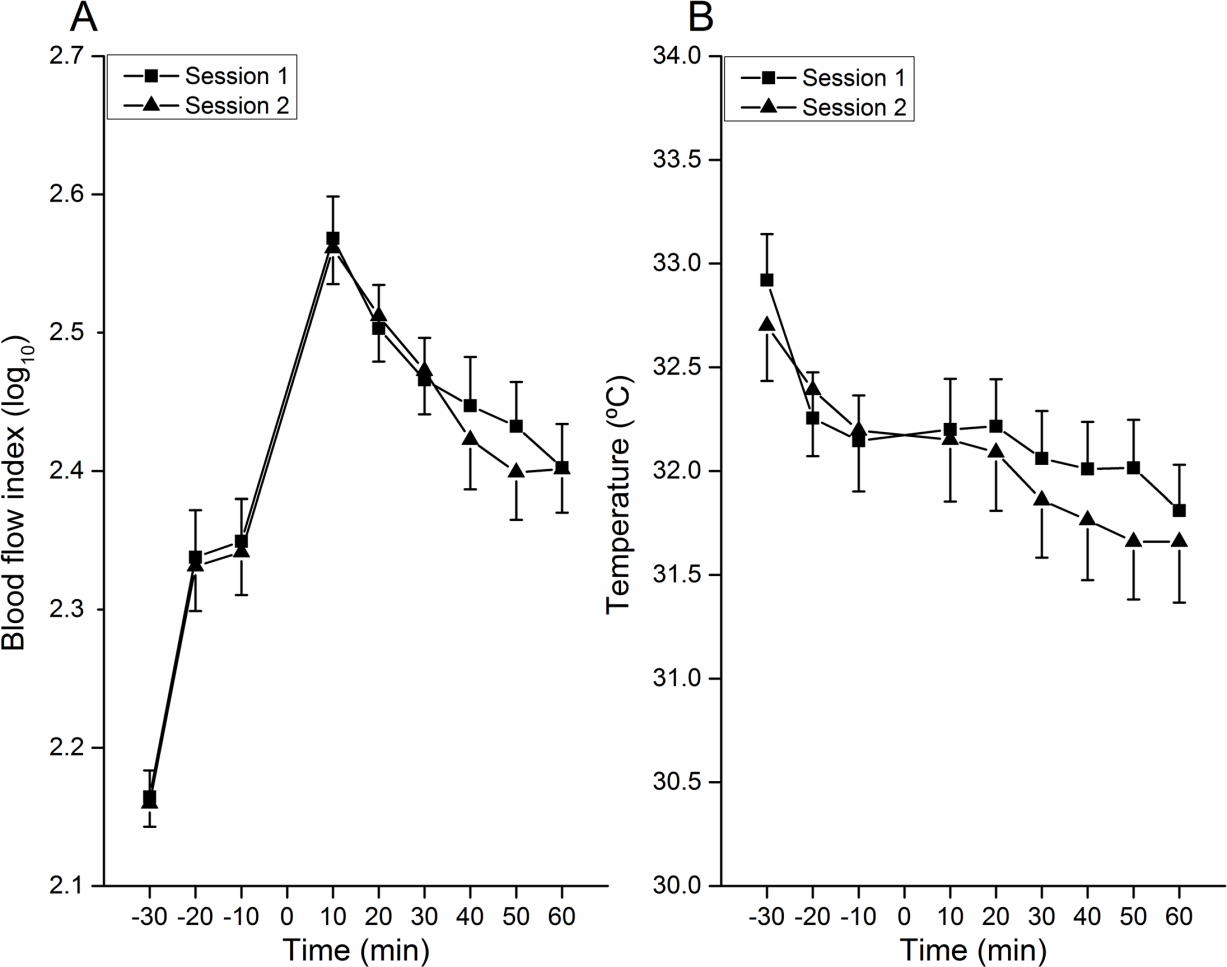
Neurogenic inflammation. A. SBF changes caused by 10 Hz CES. An increase of SBF occurred 10 min after CES. Thereafter it declined. No difference was found between the two sessions. B. ST changes caused by 10 Hz CES. ST was found to be declined along the observation period when this decline was temporarily stopped 10 min and 20 min into the postconditioning period. No difference was found between sessions.

No difference was found between the two sessions for the superficial blood flow changes (F = 0.131, p = 0.721). The superficial blood flow was found to significantly increase after CES where after it gradually returned to baseline values (time effect, F = 60.808, p<0.01). The highest superficial blood flow was observed 10 min postCES (p<0.0.05, Bonferroni-Holm) (Fig 3*A*). No interaction was found between session and time factors.

The average skin temperature was found to decline throughout the observation period (time effect, F = 19.092, p<0.01), i.e., the skin temperature preCES was higher than postCES, and the skin temperature kept on decreasing in the postCES period (p<0.05, Bonferroni-Holm) (Fig 3*B*). However, no difference was found between 10 min preCES, 10 min postCES, and 20 min postCES (Fig 3*B*). No difference was observed between sessions (session effect, F = 0.298,p = 0.591), and no interaction effect was found between session and time factors.

### Heterotopic Light-Stroking Stimuli

**Fig 4:**
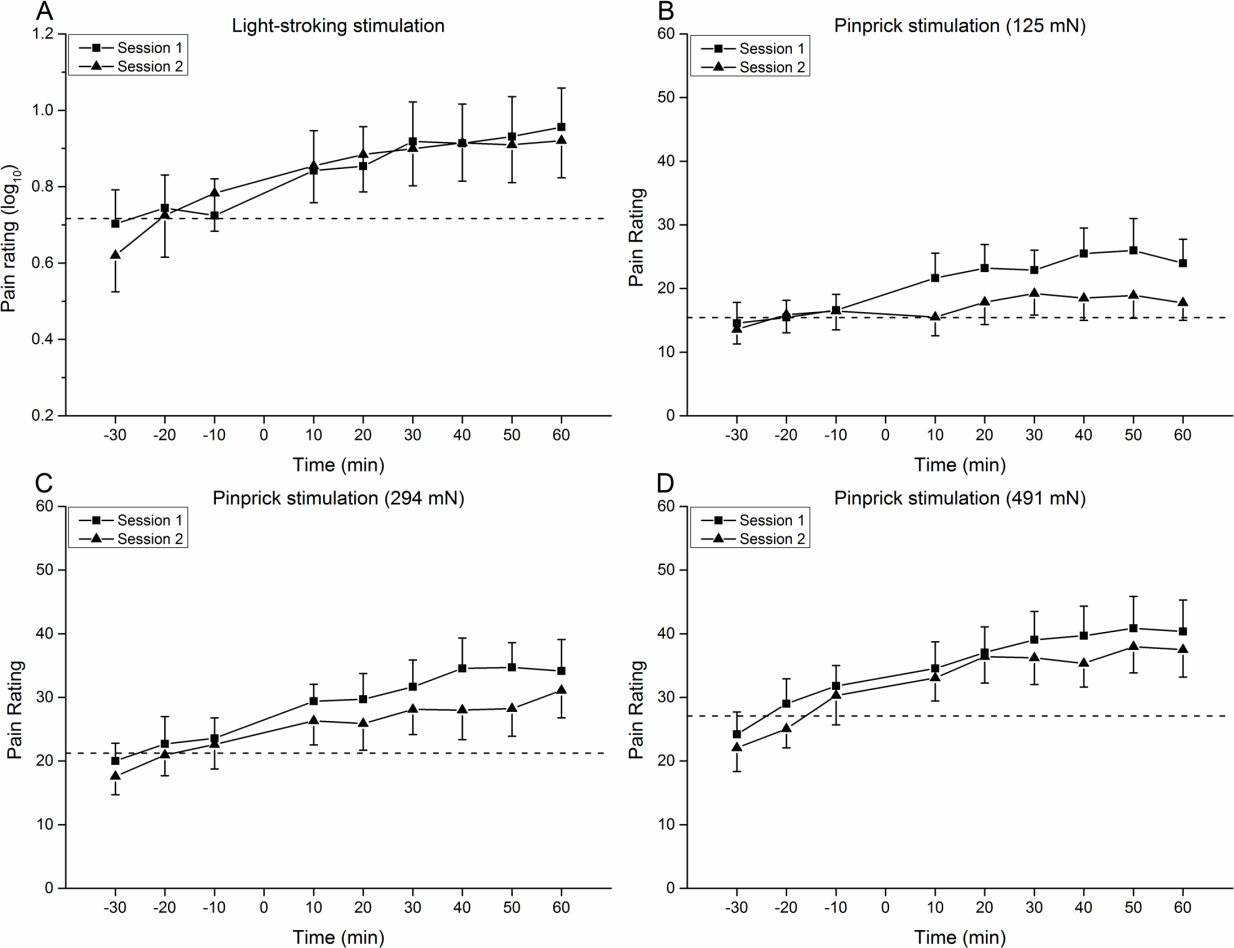
Heterotopic effect of 10 Hz CES on pain perception by mechanical stimuli. A. Light-stroking stimulation. The perception intensity of stroking stimuli in the area around the conditioned site increased after the CES until reaching the plateau about 30 min in the post conditioning period both in two sessions. B,C,D. Weight-calibrated pinprick stimuli. Perception intensities of all pinprick stimulators around the conditioned site increased after the CES which lasted at least one hour. Pinprick perception intensities in session one were higher than in session two for 125 mN and 294 mN stimulators and no difference was found for 491 mN stimulator between sessions. The dashed lines indicate the average perception intensity ratings at three time points before the 10 Hz CES.

The perception intensity of light-stroking stimuli adjacent to the conditioned site increased after the CES (p<0.05, Bonferroni-Holm) with an average increase of 25% in session one from 0.72 (preCES, log_10_; 7.6 in raw data) to 0.9 (postCES, log_10_; 12.5 in raw data) and 27% in session two from 0.71 (preCES, log_10_; 8.1 in raw data) to: 0.9 (postCES, log_10_; 12 in raw data), respectively. The stroking perception intensity increased until the end of the observation period (time effect, F = 20.836, p<0.01), i.e., the perception intensity 10 min postCES was lower than 30 min and 50 min (p<0.05, Bonferroni-Holm) (Fig 4*A*). However, no difference was found between the two sessions (session effect, F = 0.056, p = 0.816). No interaction effect was found between session and time factors.

### Heterotopic Pinprick Stimuli

The area adjacent to the conditioned site showed an increased pinprick sensitivity after the 10 Hz CES. For the 125 mN pinprick testing, the perception intensity at 30 min preCES was lower than at 30 min and 40 min postCES, and the perception intensity at 20 min preCES was lower than 40 min postCES (time effect, p<0.05, Fig 4*B*). The pain intensity increased by 54% (session one) and 17% (session two) in the postCES period, respectively. For the 294 mN pinprick testing, the perception intensity increased after CES, which lasted at least one hour (time effect, p<0.05, Fig 4*C*). The pain intensity increased by 47% (session one) and 26% (session two) in the postCES period, respectively. For the 491 mN pinprick testing, an increase of the perception intensity was found after CES, i.e., the intensities at 30 min and 20 min preCES were lower than at all the later time points; the intensities at 10 min preCES were lower than at 20 min, 30 min, 40 min, 50 min, and 60 min postCES; the intensity at 10 min postCES was lower than at 50 min postCES (time effect, p<0.05, Fig 4*D*). The pain intensity increased by 36% (session one) and 40% (session two) in the postCES period, respectively. The perception intensities in session one were higher than session two for 125 mN and 294 mN pinprick stimulation (session effect, p<0.01), however, no difference was found between session one and session two for the 491 mN pinprick stimulation.

### Homotopic Single Electrical Stimulation

**Fig 5:**
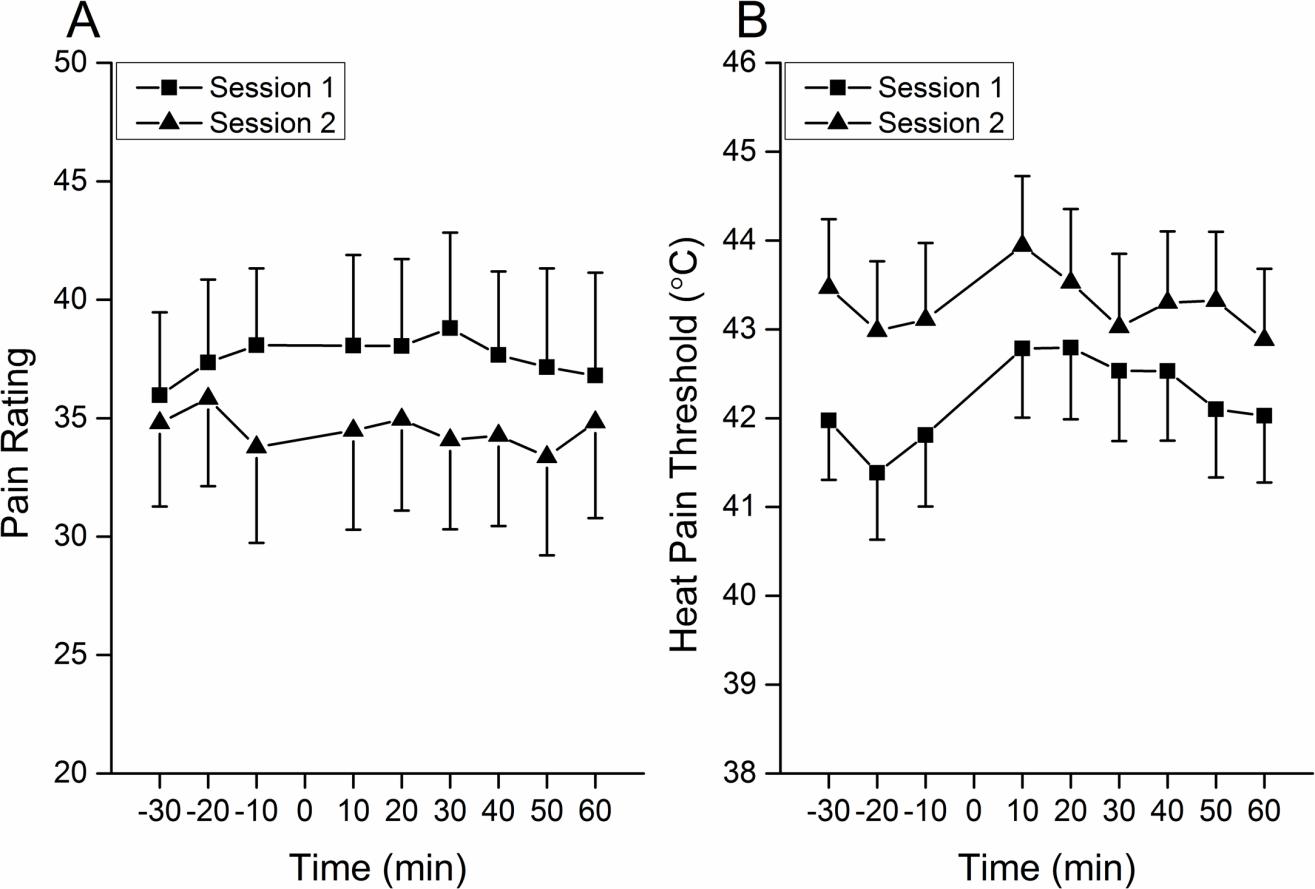
Homotopic effect of 10 Hz CES on pain perception. A. Pain intensity evoked by SES. No changes were found after 10 Hz CES for the perception intensity of SES. No differences were found between the two sessions. B. HPT assessment. The HPT increased after 10 Hz CES (10 min and 20 min in the post conditioning period) then gradually returned to the baseline. The HPT in session one was higher than in session two.

No statistically significant differences were found after CES for the perception intensity of SES (time effect, F = 0.355, p = 0.863). No differences were found between the two sessions (session effect, F = 3.037, p = 0.098) (Fig 5*A*). In addition, no interaction effect was found between session and time factors.

### Heat Pain Threshold

The HPT was increased after the CES and then gradually returned to baseline (time effect, F = 4.24, p<0.01). The HPT 20 min preCES was lower than 10 min and 20 min postCES (p<0.05, Bonferroni-Holm); the HPT 10 min preCES was lower than 10 min postCES (p<0.05, Bonferroni-Holm); the HPT 10 min postCES was higher than 30 min and 60 min postCES (p<0.05, Bonferroni-Holm) (Fig 5*B*). The HPT in session one was higher than in session two (session effect, F = 11.591, p<0.01; Fig 5*B*).

### Reliability Analysis and Sample Size Estimation

**Fig 6:**
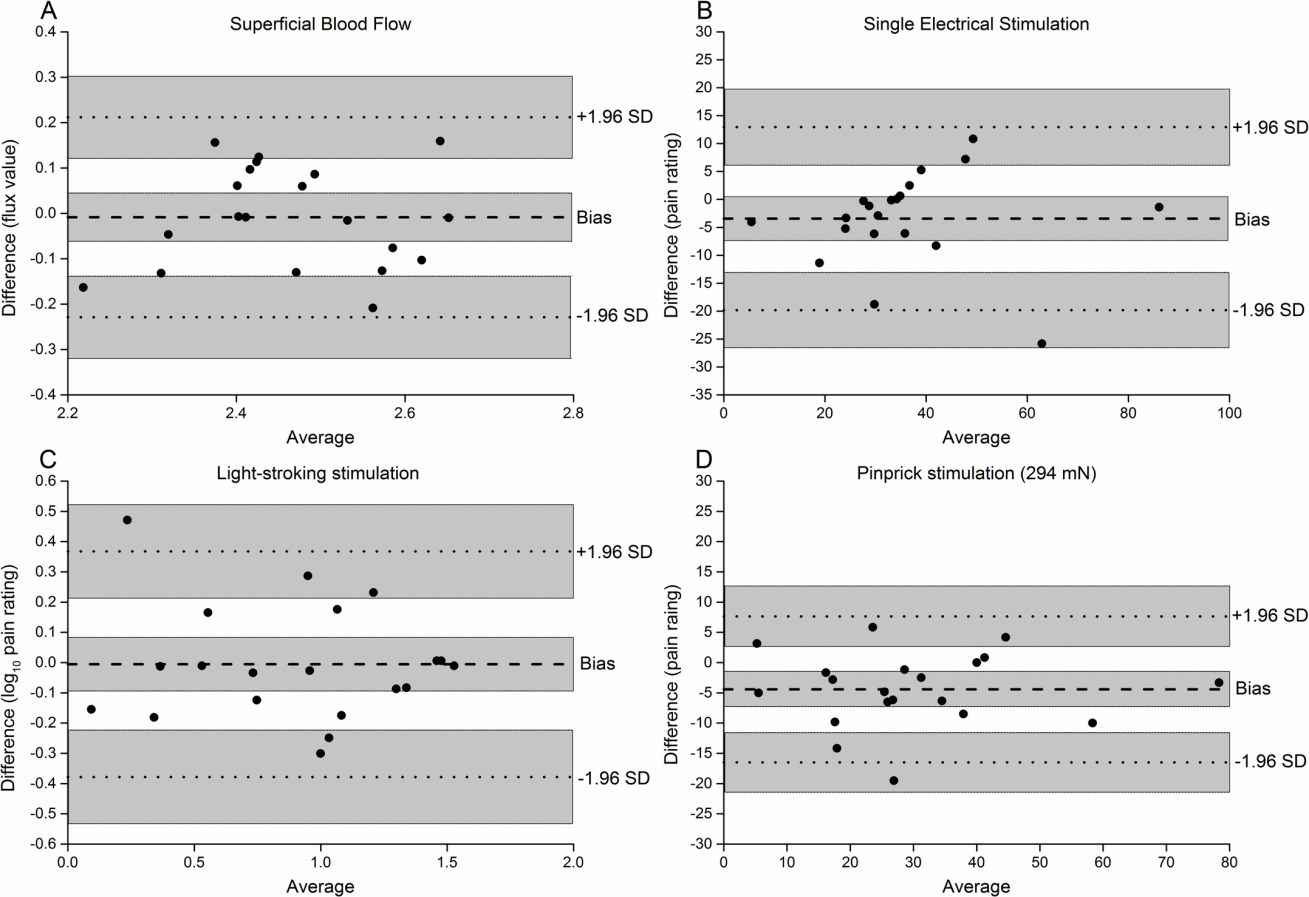
Bland-Altman plots for the effects after 10 Hz CES. Assessments were performed on the SBF, homotopic pain perception to SES, and heterotopic pain perception to light-stroking and pinprick stimulation (294 mN). The dashed line indicates the bias between sessions, whereas the dotted lines indicate the limits of agreement calculated as ±1.96×the standard deviation (SD) of the differences between measurements for the two sessions. Shaded areas indicate the confidence intervals of mean difference and limits of agreement.

The within session and between session ICC and CV are presented in Table 1, and the Bland-Altman plots are presented in Fig 6. The higher weight pinprick stimulation has a higher reliability compared with lower weight pinprick stimulation (see Table 1). Based on the Bland-Altman plots, the data of SBF, perception ratings to SES, and light-stroking stimuli are homoscedastic because the level of equality is within the CI of mean difference for each measurement. Whereas bias of the perception ratings to pinprick stimulation (294 mN) between sessions is significant as the level of equality is not inside the CI. According to the CI of limits of agreement (shaded area, Fig 6), the sampling of this study was acceptable. The estimated sample sizes for crossover and parallel study designs are shown in Table 1. Compared to crossover study designs, parallel study designs need more subjects. This means that if a certain drug or treatment is able to depress the vascular and/or sensory outcome measures by 30% induced by 10 Hz CES, more subjects will be needed for parallel study designs to test the proposed hypothesis.

**Table 1:** Reliability and sample size estimation of superficial blood flow, homotopic and heterotopic pain perception in 10 Hz CES paradigm.

Assessment measure		Sample Size		CV(%)		ICC(95% confidence interval)	
		Crossover	Parallel	Within session	Between session	Within session	Between session
Superficial blood flow		3	13	3.3	3.0	0.791 (0.701–0.868)	0.617 (0.493–0.717)
Light-stroking		2	33	8.4	12.6	0.964 (0.945–0.979)	0.885 (0.839–0.918)
Pinprick	125 mN	33	184	28.4	29.6	0.823 (0.744–0.889)	0.776 (0.693–0.838)
	294 mN	17	111	21.6	22.2	0.848 (0.778–0.906)	0.817 (0.747–0.869)
	491 mN	6	54	14.4	13.9	0.903 (0.854–0.941)	0.876 (0.827–0.912)
Single electrical stimulation		634	11310	10.2	13.9	0.944 (0.914–0.966)	0.850 (0.791–0.893)

The intra-class correlation coefficient (ICC) and coefficient of variation (CV) were calculated within and between sessions. The 95% confidence intervals of ICC were also shown. The sample sizes were estimated for potential crossover and parallel designed drug testing studies.

## Discussion

For the first time, the present study 1) investigated the reliability of a set of standard outcome measures assessing 10 Hz CES induced neurogenic inflammation and pain sensitization, and 2) estimated the sample sizes for future pharmacological studies aiming to get a 30% recovery of 10 Hz CES induced effects in crossover and parallel study designs. Sample size estimation is the basic step before starting studies using the 10 Hz CES induced neurogenic pain sensitization model. Reproducible assessment methods combined with an adequate sample size (number of subjects) can help to establish a reliable study design. The outcome measures of neurogenic inflammation and pain sensitization induced by the 10 Hz CES in the present study showed acceptable reliability, especially for application in crossover study designs.

### Features of 10 Hz CES

In the present study, the pain intensity during the 10 Hz CES process was high for 20 s then gradually decreased. The declining pain sensation during the 50 s conditioning process may be due to habituation, i.e., subjects gradually got used to the stimulus when that stimulus is applied repeatedly [18,37]. 10 Hz electrical stimulation paradigm was applied to mimic the low frequency discharge of C-fiber nociceptors during neuropathic or inflammatory pain conditions. During neurogenic or inflammatory conditions, sustained low frequency C-fiber barrage of the central nervous system may contribute to hyperalgesia [19,38–40]. In fact, C-fiber nociceptor discharge is able to follow low frequency (1–10 Hz) electrical stimulation [41]. The pain perception intensity during the conditioning process in session one was higher than during session two whereas the SF-MPQ scores and PPI were not different between the two sessions. The PPI is an average value which subjects gave for rating the pain intensity during the conditioning process. These results indicate that the overall pain experience for 10 Hz CES process is relatively stable between sessions. However, the systematic error, i.e. a learning effect, is likely to contribute to the session difference in rating the pain during the conditioning process. It is likely that subjects in session two perceived less pain as a result of their prior experience in session one [29].

### Neurogenic Inflammation–Vascular Outcome Measures

Peptidergic nerve endings, mainly C-fibers, after being activated by nociceptive electrical stimulation can release neuropeptides, e.g., substance P, calcitonin gene-related peptide [42]. These substances induce neurogenic inflammation as a consequence of capillary vasodilatation, plasma extravasation, attraction of macrophages, or degranulation of mast cells [43–45]. In the present study, the SBF significantly increased after 10 Hz CES most probably indicating the activation of these C-fiber nociceptors [10]. It took one hour for the blood flow to return to the level 10 min before CES. This allows time for observing potential drug effects on neurogenic superficial blood flow changes. The average skin temperature at the site of the blood flow flare, however, showed a declining tendency during the whole observation period. One possible reason could be that exposing the arm for one full hour could lead to gradually lower skin temperature and thereby less superficial blood flow. This will eventually also lead to lower skin temperature. However, the decline of skin temperature was halted for 20 minutes after 10 Hz CES which may indicate that 10 Hz CES indeed induced an increase in skin temperature for a short time period possibly counteracting the skin cooling. As a consequence, the skin temperature kept stable for 20 minutes after CES. Therefore, the skin temperature may not be a good indicator for neurogenic inflammation induced by the 10 Hz CES paradigm as it appears to be affected by surrounding circumstances.

A reliable assessment of neurogenic inflammation in cutaneous hyperalgesia models is extremely important as it is related to the extent of irritation on the peripheral afferents [44,46,47]. Furthermore, it can be used for assessment of drug effect [48–50]. In previous studies, the SBF has been used as an indicator for measuring neurogenic inflammation in many neurogenic hyperalgesia models as drugs may interact with peripheral afferents affecting the release of neurogenic transmitters [28,51,52]. From ICC values (ICCwi = 0.79, ICCbt = 0.62) and sample sizes estimation (Ncr = 3, Np = 13) in the present study, SBF has good reliability for measuring the neurogenic inflammation both in crossover and parallel study designs. The Bland-Altman plot showed that the level of equality was within the CI indicating acceptable agreement between two sessions. Hence, it is recommended to include this parameter when using the 10 Hz CES paradigm in potential drug testing studies.

### Heterotopic Pain-LTP

In the present study, pain ratings to pinprick stimuli (heterotopic hyperalgesia) and light stroking (dysesthesia) around the conditioned site were found to increase after 10 Hz CES. The increased perception ratings lasted until the end of the observation period (one hour). In previous studies, high frequency, repetitive, electrical stimulation of primary nociceptive C-fibers has led to dynamic mechanical allodynia and mechanical hyperalgesia surrounding the conditioned area [10,12,15]. TRPV1-positive C-fibers (major contribution) and TRPV1-positive A-fibers (minor contribution) were found to be the main inducers of heterotopic pain-LTP [53]. Furthermore, TRPV1-negative A-fibers were found to be the main afferents for mediating secondary pinprick hyperalgesia, but without taking effect in the induction of heterotopic LTP-like pain amplification [53]. In the present study, the perception intensity to non-painful pinprick stimulation (125 mN) also increased after 10 Hz CES, and therefore, both nociceptive and non-nociceptive pinprick neurotransmission seem to be facilitated by CES. Furthermore, our results support the notion that heterotopic pain-LTP reflects central mechanisms, but not peripheral mechanisms [54]. Firstly, the pinprick stimuli located outside of the blood flow flare indicating no direct influence from sensitized peripheral nociceptors due to the inflammatory mediators [55]. Secondly, the neurogenic inflammation indicators and the sensory changes had different time courses; the decrease in superficial blood flow after 10 Hz CES did not follow the increase in pinprick perception. Furthermore, it took 30 min for the pain ratings to light-stroking stimuli to reach a plateau which is also not synchronized to the vascular findings.

Regarding the measures of heterotopic pinprick hyperalgesia, the non-painful (125 mN) and mild painful (294 mN) stimulators in session one were higher than in session two. In addition, as shown in the Bland-Altman plot, the level of equality was not within the CI of the mean difference, which indicates a bias between sessions. For the painful (491 mN) stimulus, however, no significant difference was observed between the two sessions. Moreover, the most reliable results were also obtained with the heaviest pinprick stimulus (491 mN) for crossover (ICCwi = 0.90, Ncr = 6) and parallel (ICCbt = 0.88, Np = 54) study designs. The higher weight of the pinprick stimulus, the higher ICC values and smaller sample size were shown. Therefore, the painful pinprick method is a reliable indicator for the facilitation of Aδ-fiber pathway mediating heterotopic pinprick hyperalgesia. The session effect found for 125 mN and 294 mN pinprick testing may indicate that the subjects were more familiar with the experimental process, and therefore responded with smaller pain ratings (learning effect) [28]. The pain perception rating for light-stroking was reliable (ICCwi = 0.96, ICCbt = 0.89) with acceptable sample sizes (Ncr = 2, Np = 33) for measuring the pain perception at the facilitated non-nociceptive A-β fiber pathway both in crossover and parallel study designs.

### Homotopic Pain-LTP

In the present study, the pain perception to SES at the conditioned site did not increase after 10 Hz CES. This indicates the absence of homotopic pain-LTP. In van den Broeke’s study (2012), the pain intensity of SES did also not increase after 100 Hz CES in both conditioned and unconditioned skin sites, despite the coexistence of enhanced event-related potentials in the central nervous system [18] while a declining perception intensity was also observed in another study only with a minor change in the homotopic pain [17]. This was hypothesized to be caused by 1) the counter effects of LTP and long-term depression which resulted from the concurrent activation of C-fiber and A-δ fiber pathways by conditioning electrical stimulation, respectively [14]; 2) the habituation because of repetitive electrical stimuli [37]; 3) the hypoesthesia which can be induced by 20 Hz continuous CES at C-fiber strength [56]; or 4) a technical reason that the repositioning of the electrode may mask the pain amplification resulting in a statistical type-2 error in the present study. The technical reason cannot be avoided in the present study. Because of the measurement of the neurogenic inflammation, the electrode had to be removed and then placed back to the same skin area for the sensory testing. Reposition of the electrode could also change the impedance between the skin and electrode, which may influence the stimulation intensity.

The HPT was found to increase after 10 Hz CES, which indicates that heat pain hyperalgesia was also absent which was in agreement with the observations by Lang et al. (2007) for 100 Hz conditioning stimulation [13]. This might reflect the same mechanisms for the absence of increased pain perception to single electrical stimulation. Thermal hyperalgesia at the stimulation/injury area is a typical feature of primary hyperalgesia and has been demonstrated to be largely due to the primary afferents sensitization [57]. Therefore, the absence of heat hyperalgesia at the conditioned area in humans indicates a lack of peripheral sensitization in the 100 Hz and 10 Hz CES models; alternatively, the HPT measurement employed in the present study is not a good indicator for testing the heat hyperalgesia compared with suprathreshold heat stimuli [16,58]. The LTP in the C fiber pathway is both NMDA receptor and AMPA receptor-dependent [59,60]. The inhibitors of calcium/calmodulin dependent protein kinase II, protein kinase A, protein kinase C, and phospholipase C have been shown to prevent induction of spinal LTP in the signal transduction pathways indicating that LTP induction is calcium dependent [19,61,62]. LTP induction also relies upon other molecular players such as substance P and the neurokinin 1 receptor [19]. In human studies, low-dose of ketamine has been reported to prevent hyperalgesia at the conditioned site in the 100 Hz CES paradigm, but not able to affect secondary hyperalgesia or allodynia at adjacent skin areas [63]. In the present study, the pain perception intensity to SES for testing the homotopic pain-LTP showed an acceptable, relative reliability both in within and between sessions (ICCwi = 0.94, ICCbt = 0.85). However, the minor effect of 10 Hz CES on the homotopic pain amplification observed in the present study led to a large number of subjects required in potential crossover and parallel studies. This indicates that additional future studies are needed to understand the complex homotopic pain amplification mechanisms.

## Conclusions

The present study showed that in the 10 Hz CES paradigm, the induced neurogenic inflammation and heterotopic mechanical hyperalgesia outcome measures are reliable for a period of at least one hour and between two CES sessions with at least one week interval. Assessment of superficial blood flow is highly reliable and recommended for assessment of neurogenic inflammation in both potential crossover and parallel drug testing studies. In potential crossover drug testing studies, heavy painful (491 mN) pinprick stimulation reliably assessed heterotopic mechanical hyperalgesia Assessment of dysesthesia by light-stroking stimuli showed an excellent reliability in both potential crossover and parallel drug testing studies. However, the absence of homotopic hyperalgesia indicates the complex mechanisms when applying different patterns of CES, which need further research. In conclusion, it is important to select adequate assessment tools and study designs with good reliability before conducting experiments to test drug effects. The results from this study can be an essential link between animal and human nociception studies and make the 10 Hz CES paradigm an alternative for inducing pain LTP in healthy humans.
